# Inhibition of TRPA1 Ameliorates Periodontitis by Reducing Periodontal Ligament Cell Oxidative Stress and Apoptosis via PERK/eIF2*α*/ATF-4/CHOP Signal Pathway

**DOI:** 10.1155/2022/4107915

**Published:** 2022-06-10

**Authors:** Qian Liu, Shujuan Guo, Yanli Huang, Xiuqun Wei, Li Liu, Fangjun Huo, Ping Huang, Yafei Wu, Weidong Tian

**Affiliations:** ^1^State Key Laboratory of Oral Diseases, & National Clinical Research Center for Oral Diseases, & National Engineering Laboratory for Oral Regenerative Medicine, West China School of Stomatology, Sichuan University, Chengdu 610041, China; ^2^Engineering Research Center of Oral Translational Medicine, Ministry of Education, West China School of Stomatology, Sichuan University, Chengdu 610041, China; ^3^Department of Periodontics, West China Hospital of Stomatology, Sichuan University, Chengdu 610041, China; ^4^Department of Oral and Maxillofacial Surgery, West China Hospital of Stomatology, Sichuan University, Chengdu 610041, China

## Abstract

**Objective:**

In periodontitis, excessive oxidative stress combined with subsequent apoptosis and cell death further exacerbated periodontium destruction. TRPA1, an important transient receptor potential (TRP) cation channel, may participate in the process. This study is aimed at exploring the role and the novel therapeutic function of TRPA1 in periodontitis.

**Methods:**

Periodontal ligament cells or tissues derived from healthy and periodontitis (PDLCs/Ts and P-PDLCs/Ts) were used to analyze the oxidative and apoptotic levels and TRPA1 expression. TRPA1 inhibitor (HC030031) was administrated in inflammation induced by *P. gingivalis* lipopolysaccharide (*P.g.*LPS) to investigate the oxidative and apoptotic levels of PDLCs. The morphology of the endoplasmic reticulum (ER) and mitochondria was identified by transmission electron microscope, and the PERK/eIF2*α*/ATF-4/CHOP signal pathways were detected. Finally, HC030031 was administered to periodontitis mice to evaluate its effect on apoptotic and oxidative levels in the periodontium and the relieving of periodontitis.

**Results:**

The oxidative, apoptotic levels and TRPA1 expression were higher in P-PDLC/Ts from periodontitis patients and in *P.g.*LPS-induced inflammatory PDLCs. TRPA1 inhibitor significantly decreased the intracellular calcium, oxidative stress, and apoptosis of inflammatory PDLCs and decreased ER stress by downregulating PERK/eIF2*α*/ATF-4/CHOP pathways. Meanwhile, the overall calcium ion decrease induced by EGTA also exerted similar antiapoptosis and antioxidative stress functions. In vivo, HC030031 significantly reduced oxidative stress and apoptosis in the gingiva and periodontal ligament, and less periodontium destruction was observed.

**Conclusion:**

TRPA1 was highly related to periodontitis, and TRPA1 inhibitor significantly reduced oxidative and apoptotic levels in inflammatory PDLCs via inhibiting ER stress by downregulating PERK/eIF2*α*/ATF-4/CHOP pathways. It also reduced the oxidative stress and apoptosis in periodontitis mice thus ameliorating the development of periodontitis.

## 1. Introduction

Periodontitis, the leading cause of tooth loss and disability among adults influencing as many 3.5 billion global population [1], is characterized by irreversible hard and soft tissue loss and unstable periodontal homeostasis [2], which closely relates to oxidative stress (OS) such as activated reactive oxygen species (ROS) [3, 4]. Oxidative stress exacerbates lipid peroxidation, protein misfolding and dysfunction [5], DNA damage [6], and organelle stress [7] like mitochondria or endoplasmic reticulum (ER) to accelerate defective cell differentiation and cell apoptosis or necrosis. In addition, oxidative stress and subsequent apoptosis are critical in the development of oxidative stress-associated disorders and become the target of therapeutic interventions [8–10]. Similarly, in periodontitis, the hypoxic subgingival environment triggering aberrant apoptosis would contribute to the pathogenesis by enabling persistent infection and aggravating host immune responses, which would cause an imbalance between resorption destruction and mineralization reconstruction of periodontal tissue [11]. A study reported that both oxidative stress and apoptosis appeared earlier before periodontitis initiation [11]. In periodontitis, increased OS biomarkers and apoptosis markers were detected in saliva, gingival crevicular fluid, and plasma [12–16]. After periodontal treatment, these OS or apoptosis biomarkers were significantly decreased [9, 17, 18] and antioxidants were increased [19]. ROS overproduction and aberrant apoptosis promoted alveolar bone loss during the development of periodontitis [20]. Subsequently, protecting periodontal tissues or cells against various factor-induced oxidative stress and apoptosis is a potential solution to postpone periodontitis initiation and development. Modestly preventing or removing excessive ROS or apoptosis plays a crucial role in regulating the microenvironment of periodontitis and provides favorable conditions for periodontal homeostasis [15, 21, 22]. Thus, successfully decreasing oxidative stress and avoiding oxidative stress-induced apoptosis are likely therapeutic targets in periodontitis.

Transient receptor potential ankyrin 1 (TRPA1) is of special attention and interest for being the most nonselective permeable Ca^2+^ channels in the transient receptor potential cation channels superfamily and plays an important role in oxidative stress, apoptosis, and inflammation of respiratory disorders [23–25], cardiovascular diseases [26], chronic arthritis [27], retinal damage [28] diabetes, and obesity [29], and parts of its antagonists were used as an novel treatments for these disease [24, 25]. It significantly attenuated ROS production [30], hypoxia-induced apoptosis [31], inflammation proinflammatory cytokines [32] mainly via blocking Ca^2+^ influx, and subsequent ER stress or mitochondrial oxidative stress [23]. Moreover, one of TRPA1 inhibitor, GRC-17536 has been used in phase II clinical trials in the treatment of respiratory disorders and diabetic peripheral neuropathy (NCT01726413) [24, 25]. Thus, TRPA1 was considered an attractive therapeutic target of antioxidative, antiapoptosis, and anti-inflammation for the treatment of related diseases.

TRPA1 is expressed in various dental-derived cells, such as human odontoblast-like cells [33–35], human dental pulp cells [36], human dental pulp fibroblasts [37], and periodontal ligament cells (PDLCs) [38], and plays a pivotal role in inflammatory pain and mechanical hyperalgesia [39]. Mechanical force has been reported to produce oxidants in PDLCs, the oxidant-sensitive channel TRPA1-mediated orthodontic force-induced oxidative stress and related pain [38]. The activation of transient receptor potential (TRP) channels increased Ca^2+^ in human PDLCs and further induced IL-6 and IL-8 expression under thermal stress [40]. The role of TRPA1 in periodontal ligament tissues and cells depicted its thermo- and mechanosensitive properties in normal periodontium [41]. Other TRPs, such as TRPV4, TRPV1, and TRPM3, are critical in alveolar bone remodeling via regulating RANKL/OPG signaling pathway under mechanical stress without periodontitis [42–44], while in periodontitis, little is known about the role of TRPs and the expression and functions of TRPA1 in periodontitis are rarely studied.

Therefore, this study mainly investigated the expression pattern of TRPA1 in periodontitis, explored its effect on the oxidative stress and apoptosis of PDLCs under inflammation and the precise molecular mechanism involving ER stress in vitro and in vivo, and further found its potential application in the treatment of periodontitis.

## 2. Materials and Methods

All experiments with human periodontal ligament cell (PDLC) cultures and mice in this study were approved and registered by the Animal Care and Use Committee and Ethics Committee of West China College of Stomatology, Sichuan University (WCHSIRB-D-2021-511 and WCHSIRB-D-2021-55).

### 2.1. Isolation and Purification of PDLCs and P-PDLCs

Extracted human premolars due to orthodontic treatment used for PDLCs isolation were donated by 11 systemic healthy volunteers aged 15-45 years (5 males and 6 females) with good oral health. Unpreservable periodontitis teeth used to isolate P-PDLCs were obtained from systemic healthy periodontitis patients (25-45 years, 4 males and 4 females). All donors were requested to sign the informed consent form. P-/PDLC isolations were described in detail in previous protocols [45]. Both PDLCs and P-PDLCs at passages 2-4 were used in experiments.

### 2.2. Animal Experiments

Seven-week-old male C57BL/6J mice were used for experimental periodontitis modeling as previously described [46]. After a week, the right maxillaries (*N* = 5) were scanned by micro-CT to evaluate the completion of the experimental mouse periodontitis model. The in vivo study contains 4 groups: (1) negative control (healthy mice with solvent only), (2) periodontitis group (initially ligated mice with solvent only), (3) HC1 group (initially ligated mice with 10 mg/kg HC030031), and (4) HC3 group (initially ligated mice with 30 mg/kg HC030031). All mice were intraperitoneally administrated and weighted every day for a week. The gingiva around the molars were collected for Western blot. The maxillaries were fixed for micro-CT evaluation and histological staining. The total serum was prepared for total CRP and oxidative stress level detection.

### 2.3. Real-Time Quantitative PCR (RT-qPCR)

Total RNA of PDLCs and P-PDLCs or mouse gingiva was extracted (RNAiso plus® (Takara, Japan)) and was reverse transcribed using RNA reverse transcription kit (Vazyme, China). Real-time quantitative PCR was performed on the Real-Time PCR System (Life Technologies^TM^, Singapore) by using SYBR PrimeScript^TM^ RT-PCR Kit (Takara, Japan). The 2-*ΔΔ*Ct method was used. Parallel triplicates were prepared each time. The primers used were shown in supplementary information (Table S1).

### 2.4. Western Blot

The total protein of PDLCs and P-PDLCs or mouse gingiva was extracted, and 20 *μ*g protein of each sample was separated and blotted onto a polyvinylidene fluoride membrane (Millipore, USA). Then, membranes were blocked and incubated with primary antibodies including anti-actin (Abcam, UK; ab3280, 1 : 10000), anti-cleaved Caspase-3 (Cell Signaling Technology, USA; #9664, 1 : 1000), anti- PPAR*γ*2 (Abcam, UK; ab45036, 1 : 1000), anti-HSP70 (Zen, China; 200304, 1 : 1000), anti-TRPA1 (Novus, USA; NB100-91319, 1 : 1000), anti-SOD1 (Abcam, UK; ab51254, 1 : 1000), anti-SOD2 (Santa Cruz, USA; sc-133254, 1 : 1000), anti-PERK (Cell Signaling Technology, USA; #5683, 1 : 1000), anti-CHOP (Cell Signaling Technology, USA; #2895, 1 : 1000), anti-ATF-4 (Cell Signaling Technology, USA; #11815, 1 : 1000), anti-eIF2*α* (Cell Signaling Technology, USA; #5324, 1 : 1000), and anti-p-eIF2*α* (Cell Signaling Technology, USA; #3398, 1 : 1000) at 4°C overnight. The membranes were washed and incubated with secondary antibody (1 : 10,000) and were visualized by the Image Quant Tanon-5200Multi (Tanon, China). The relative intensity of the protein was quantitatively analyzed by the ratio of actin, and all assays were repeated three times.

### 2.5. Histological, Immunohistochemical, and Immunofluorescent Analysis

Human PDL tissue (derived from 4 periodontitis and 5 nonperiodontitis with signed informed consent) was collected, fixed, and dehydrated and then prepared for frozen sections (5 *μ*m). And mouse maxillaries were fixed, decalcified by 10% EDTA for 1 month, dehydrated, and embedded in paraffin section (5 *μ*m). Hematoxylin and eosin (H&E) staining was routinely performed as previously described [46]. Immunohistochemistry (IHC) and immunofluorescence (IF) were used in this study, including primary antibodies anti-cleaved Caspase-3 (1 : 200), anti-HSP70 (1 : 200), anti-TRPA1 (1 : 200), anti-CHOP (1 : 200), and anti-PERK (1 : 200) and secondary antibodies to mice or rabbit (Zhongshanjinqiao, ZB-2305 or ZB-2301, China) and fluorescent secondary antibodies (Invitrogen, A11001 or A11008 or A21422 or A21432, USA). 1% BSA was used as the black control. Apoptotic cells in PDL tissue were labeled by the fluorescein TUNEL assay (Beyotime, C1089, China), and nuclei were stained with DAPI.

For immunofluorescence staining of PDLCs in vitro, the levels of ROS, ER stress, and mitochondrial stress were detected by fluorescent probe dihydrofluorescein diacetate (DCFH-DA, Beyotime, S0033S, China), Dihydrorhodamine 123 (MCE, 109244-58-8, USA), ER-ID (ENZO, ENZ-51026-K500, USA), and JC-1 (MCE, 3520-43-2, USA). Respectively, after 24 h posttreatment, PDLCs were washed with PBS for three times and incubated with DCFH-DA (1 : 1000) for 40 min, with Dihydrorhodamine 123 (10 *μ*M) for 20 min, with JC-1 (10 *μ*M) for 40 min, and with ER-ID (10 *μ*M) for 20 min at 37°C and nuclei were stained by DAPI. All samples were imaged under Inverted microscope (Olympus, Japan) or confocal laser scanning microscopy (Olympus, Japan) and semiquantitative analyzed by using the Image J software.

### 2.6. Flow Cytometry

After 24 h posttreatment, PDLCs were harvested and stained by using Annexin V-FITC/Propidium Iodide kit (B&D Biosciences, 556547, USA) according to the manufacturer's instructions. Next, the samples were detected with a flow cytometer (Life Technologies, USA) and analyzed with the FlowJo V10 software. All assays were repeated three times.

### 2.7. Measurement of Ca^2+^ Levels

For the measurement of Ca^2+^ levels, fura-2, AM (Invitrogen, 2256798, USA) was used. After 24 h, PDLCs were washed by PBS for three times and then stained with 2 *μ*M fura-2-AM for 45 min at 37°C and measured in a microplate reader (Thermo Fisher Scientific, USA).

### 2.8. Transmission Electron Microscope Scanning

TEM was applied to the ER and mitochondria of PDLCs as described [47]. Different groups of PDLCs were fixed by 2.5% glutaraldehyde for 15 min and collected at 4°C overnight. After fixed by 1% osmium tetroxide for 2 h at room temperature, all samples were dehydrated, embedded, and prepared for sections imaged by FEI Talos F200S ATWIN Transmission electron microscope (Hitachi, Japan, 80.0 kV). Semiquantitative analysis was performed using the ImageJ software.

### 2.9. Assay of Intracellular or Serum Oxidative Stress Levels

For the intracellular or serum oxidative stress levels, SOD kit (Beyotime, S0103, China) and MDA kit (Beyotime, S0131S, China) were applied. The total proteins of PDLCs or serum were immediately detected by using SOD or MDA kit following to the manufacturer's instructions and detected by a microplate reader (Thermo Fisher Scientific, USA). The assays were repeated three times.

### 2.10. Micro-CT Tomography Evaluation

SkyScan 1176 desktop X-ray Micro-CT system (Skyscan, Bruker) (0.5 mm filter, image, pixel size: 17.75 *μ*m, voltage: 60 kV, and electrical current: 400 *μ*A) was used to scan the molar regions of the maxillary. The exposure time was 1080 ms. Samples were scanned 1800°, with a rotation step of 0.300°. NRecon cone-beam reconstruction software and CTAn software were applied to reconstruct and analyze the data.

### 2.11. Statistical Analysis

All results were presented as the mean and standard error of the mean (mean ± SEM); all statistical analyses were performed by the GraphPad Prism V9.2 software. Student's *t*-test was used for two unpaired groups, and one-way ANOVA followed by Fisher's least significant difference (LSD) was used for multiple groups. Statistical significance was defined as *P* < 0.05.

## 3. Results

### 3.1. Periodontitis-Derived Periodontal Ligament Cells and Tissues Suffered Higher Levels of Oxidative Stress and Apoptosis

**Figure 1:**
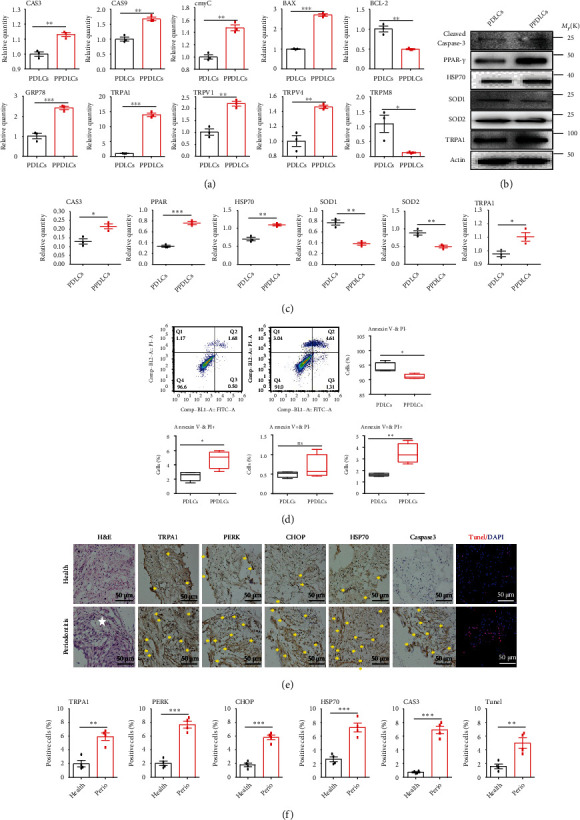
Periodontitis-derived periodontal ligament cells and tissues were at high levels of oxidative stress and apoptosis. (a) The related gene expressions of oxidative stress, apoptosis, and some of the TRP families in healthy and periodontitis-derived periodontal ligament cells (PDLCs and P-PDLCs) (*n* = 3). (b, c) Western blot and semiquantitative statistical analysis of oxidative stress, apoptosis, and TRPA1 in PDLCs and P-PDLCs. (*n* = 3). d, Flow cytometry analysis of PDLCs and P-PDLCs (*n* = 4). (e, f) H&E staining (white star represent immune cell infiltration), immunohistochemistry and immunofluorescence staining, and semiquantitative statistical analysis of periodontitis and healthy derived periodontal ligament tissues (PDLTs and P-PDLTs) (*n* = 3). Data analysis was performed by using Student's *t*-test (^∗^*P* < 0.05, ^∗∗^*P* < 0.01, and ^∗∗∗^*P* < 0.001). The data are presented as the mean ± SEM.

Periodontitis-derived periodontal ligament cells (P-PDLCs) were more prone to apoptosis and suffer higher levels of oxidative stress. On the transcriptional level, P-PDLCs expressed higher levels of GRP78, Caspase-3, Caspase-9, cymC, and Bax, but lower Bcl-2 (Figure 1(a)). Western blot results also verified that proapoptosis proteins like cleaved Caspase-3 and PPAR-*γ* were increased, and oxidative stress markers like HSP70 were highly expressed, and SOD1/2 was significantly lowly expressed in P-PDLCs (Figures 1(b) and 1(c)). Flow cytometry analysis showed that Annexin V-negative and PI-positive cells (dead cells) and Annexin V-positive and PI-positive cells (apoptosis cells) were significantly increased in P-PDLCs after 24 h culture (Figure 1(d)). H&E staining of human PDL tissue indicated that more lymphocytes and neutrophils were infiltrated locally in the periodontitis group(White star in Figure 1(e)). TUNEL-positive cells, cleaved Caspase-3, HSP70, PERK, and CHOP were highly expressed in P-PDLTs compared with healthy PDLTs (Figures 1(e) and 1(f)).

### 3.2. TRPA1 Was Highly Expressed in Periodontitis-Derived Periodontal Ligament Cells and Tissues

Typical represent subtypes of TRP superfamily (TRPA1, TRPM8, TRPV4, and TRPV1) related to inflammation and dental research were detected on the transcript level. Among TRP superfamily, TRPM8 was significantly lowly expressed in P-PDLCs, while TRPA1, TRPV4, and TRPV1 were highly expressed, and TRPA1 was with higher basal expression levels and fold changes (Figure 1(a)). Western blot results also verified TRPA1 was highly expressed in P-PDLCs (Figures 1(b) and 1(c)). Similarly, immunohistochemical staining results indicated TRPA1 was significantly highly expressed in P-PDLTs (Figures 1(e) and 1(f)).

### 3.3. LPS Induced Intracellular Calcium Concentration by Activating TRPA1 at 24 h

**Figure 2:**
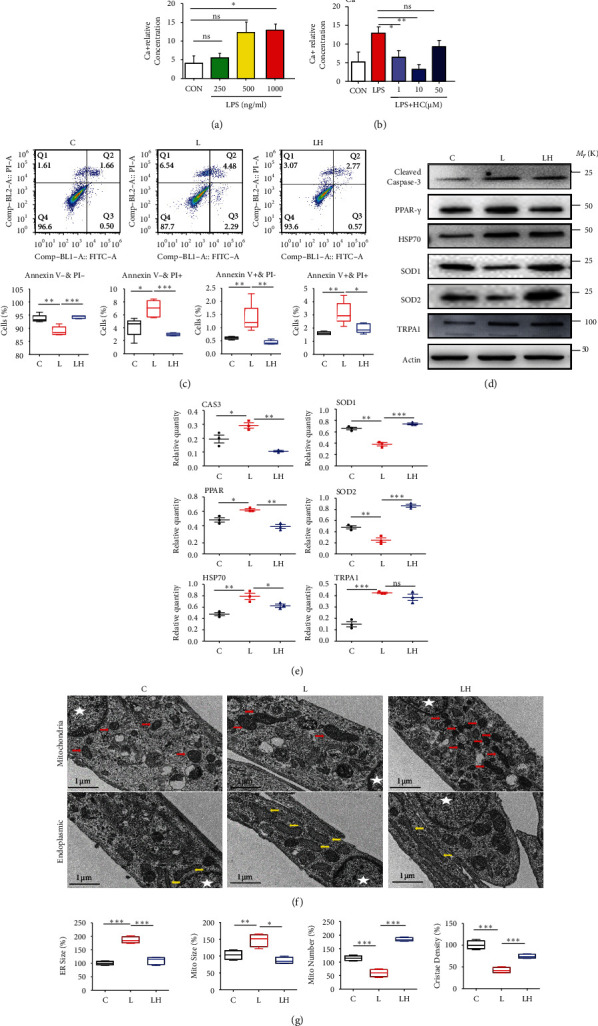
TRPA1 inhibitor HC030031 significantly ameliorated the oxidative stress and apoptosis levels of Pg.LPS-induced PDLCs. (a) P.g.LPS increased intracellular calcium ion level in a dose-dependent manner (*n* = 3). (b) Lower HC030031 concentration significantly reversed the increase of intracellular calcium ions, and 10 *μ*M HC030031 was used in the subsequent experiment (*n* = 3). (c) Flow cytometry analysis of the control group (PDLCs only, C), L group (PDLCs treated by LPS, L), and LH group (PDLCs treated by 10 *μ*M HC030031 and LPS, LH) (*n* = 4). (d, e) Western blot analysis and semiquantitative statistical analysis of oxidative stress, apoptosis, and TRPA1 proteins in in C, L, and LH groups (*n* = 3). (f) EM images showing endoplasmic reticulum (yellow arrows) and mitochondrial morphology (red arrows) of PDLCs in Ctr, LPS, and LH groups (white stars represent cell nuclei) (*n* = 4). (g) Quantification of endoplasmic reticulum size, mitochondrial size, mitochondrial number per cell (*n* = 4), and mitochondrial crista density was analyzed (>100 mitochondria). Data analysis was performed by using one-way ANOVA (^∗^*P* < 0.05, ^∗∗^*P* < 0.01, and ^∗∗∗^*P* < 0.001). Data are presented as the mean ± SEM.

*P.g.*LPS increased intracellular Ca^2+^ level in a dose-dependent manner. 1 *μ*g/ml *P.g.*LPS significantly increases intracellular Ca^2+^ and was applied to mimic the inflammatory environment (Figure 2(a)). HC030031, the TRPA1 inhibitor, significantly reverses intracellular Ca^2+^ increase. The lower concentration of HC030031 (1 *μ*M or 10 *μ*M) had a good effect on reversing intracellular Ca^2+^ increase (Figure 2(b)). 10 *μ*M was used in the subsequent experiment to ensure maximum inhibition of TRPA1 receptors and minimal impact on cell viability (Fig. S1).

### 3.4. TRPA1 Inhibitor Reduced LPS-Induced Oxidative Stress and Apoptosis in PDLCs

*P.g.*LPS induced higher levels of oxidative stress and apoptosis in PDLCs. Western blot verified that cleaved Caspase-3, PPAR-*γ*, and HSP70 increased, and SOD1/2 significantly decreased in the LPS group compared with the control group. With the treatment of HC030031, the expression of the above proteins was significantly reversed (Figures 2(d) and 2(e)). Flow cytometry analysis showed that *P.g.*LPS induced more apoptosis and dead cells, and with the treatment of HC030031, apoptosis and dead cells were reduced (Figure 2(b)). Moreover, *P.g.LPS* significantly induced the expression of TRPA1 in PDLCs, while HC030031 did not interfere TRPA1 expression in inflammation (Figures 2(d) and 2(e)).

### 3.5. TRPA1 Inhibitor Reduced LPS-Induced Endoplasmic Reticulum Oxidative Stress in PDLCs

**Figure 3:**
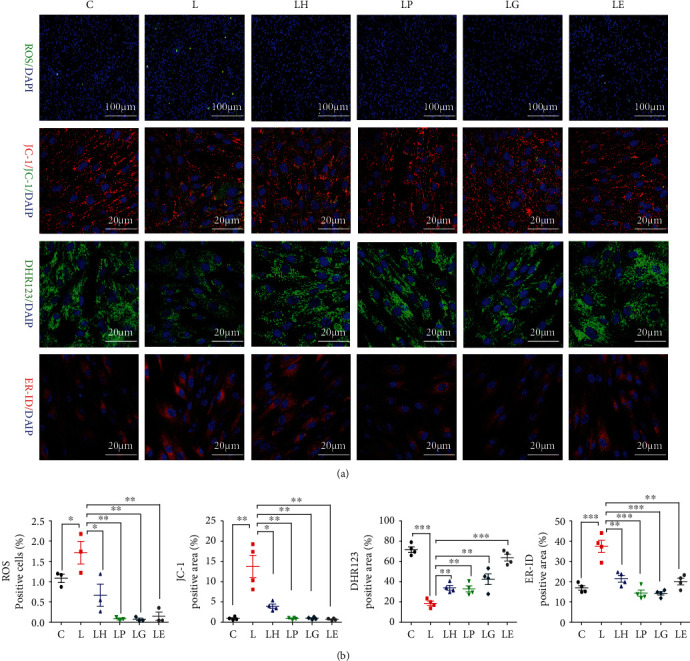
Immunofluorescence staining of ROS, JC-1, ER-ID, and DHR123 of PDLCs. (a) Immunofluorescence staining of ROS, JC-1, ER-ID, and DHR123 in C, L, LH, LP, LG, and LE groups (*n* = 4). (b) Semiquantification of immunofluorescence staining (*n* = 4). Data analysis was performed by using one-way ANOVA (^∗^*P* < 0.05, ^∗∗^*P* < 0.01, and ^∗∗∗^*P* < 0.001). Error bars represent mean ± SEM.

To further explore the precious molecular mechanism by which TRPA1 regulated oxidative stress and apoptosis in inflammation, TEM and IF staining was used to identify intracellular organelles. Endoplasmic reticulum (ER) and mitochondrial size were significantly increased in the LPS group. The mitochondrial number and cristae density, which represented the normal function and morphology of the cells, decreased significantly in the LPS group and came to a more normal level similar to the control after pretreated with HC030031 (Figures 2(f) and 2(g)). IF staining (ER-ID) demonstrated the stronger red fluorescence was observed in the *P.g.LPS* group indicating stronger ER stress, while HC030031 administration decreased red fluorescence, which indicated that ER stress was implicated (Figures 3(a) and 3(b)).

### 3.6. HC030031 Reduced Oxidative Stress and Apoptosis via the PERK/eIF2*α*/ATF-4/CHOP Pathway

**Figure 4:**
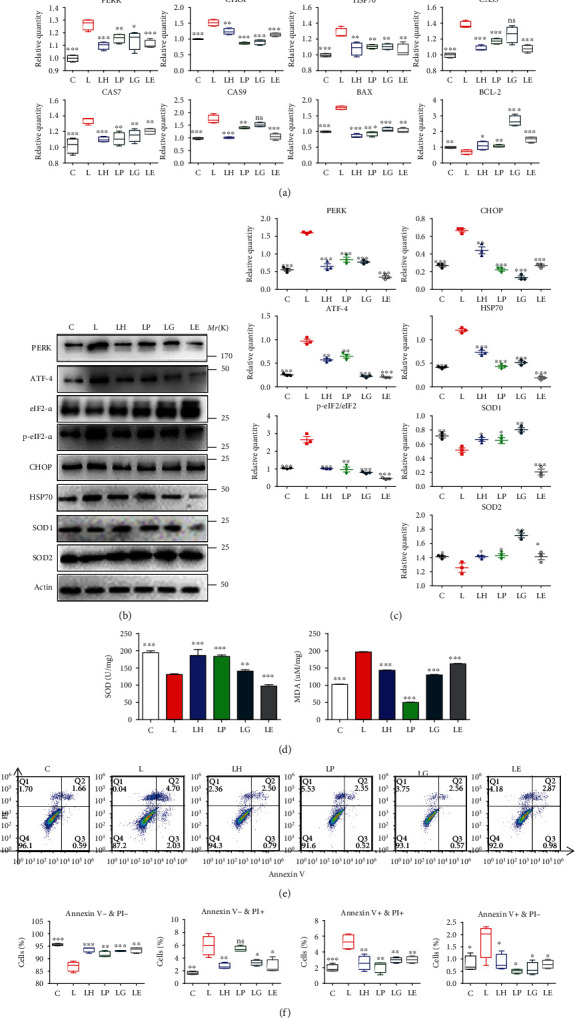
HC030031 decreased endoplasmic reticulum oxidative stress through the PERK/eIF2*α*/ATF-4/CHOP pathway. (a) The related gene expressions of PDLCs in the control group (PDLCs only, C), LPS group (PDLCs treated by LPS, L), LH group (PDLCs treated by 10 *μ*M HC030031 and LPS, LH), LP group (PDLCs treated by 10 *μ*M 4-PBA and LPS, LP), LG group (PDLCs treated by 10 *μ*M GSK2656157 and LPS, LG), and LE group (PDLCs treated by 10 *μ*M EGTA and LPS, LE) (*n* = 4). (b, c) Western blot and semiquantitative statistical analysis of PDLCs in C, L, LH, LP, LG, and LE groups (*n* = 3). (d) Total SOD and MDAs of PDLCs in C, L, LH, LP, LG, and LE groups (*n* = 4). (e, f) Flow cytometry and quantitative analysis of PDLCs in C, L, LH, LP, LG, and LE groups (*n* = 4). Data analysis was performed by using one-way ANOVA and LSD (^∗^*P* < 0.05, ^∗∗^*P* < 0.01, and ^∗∗∗^*P* < 0.001). Error bars represent mean ± SEM.

To verify the involvement of ER stress, the PERK/eIF2*α*/ATF-4/CHOP signaling pathway was detected, which is an important ER stress signaling pathway and its downstream CHOP is closely associated with inflammation and oxidative stress. After 24 h treatment of *P.g.LPS*, PERK/eIF2*α*/ATF-4/CHOP signal pathway was significantly activated, which was verified by RT-qPCR and Western blot, while the TRPA1 inhibitor pretreatment downregulated this pathway. PERK inhibitor significantly reduced PERK/eIF2*α*/ATF-4/CHOP signal pathway activation, and CHOP inhibitors significantly reduced CHOP and HSP70 in inflammation. Both inhibitors simultaneously significantly reduced oxidative stress and apoptosis of inflammatory PDLCs, which were in accordance with the results of the LH group (Figures 4(a)–4(c)).

Meanwhile, the total SOD expression was improved after treated by PERK and CHOP inhibitors, and MDAs decreased to the control level (Figure 4(d)). Flow cytometry analysis showed that both PERK and CHOP inhibitors could significantly decrease apoptosis and dead cells (Figures 4(e) and 4(f)). The LPS group showed stronger red ER-ID fluorescence, more ROS positive cells, and stronger green JC-1 staining fluorescence and weaker green DHR123 staining fluorescence, which indicated higher level of oxidative stress, while HC030031, PERK inhibitor, and CHOP inhibitor could reverse the above results (Figures 3(a) and 3(b)).

### 3.7. EGTA Also Exerted Antiapoptosis and Antioxidative Stress Functions

Since the TRPA1 inhibitor reduced intercellular calcium ions increase in inflammation, EGTA was used to trap overall calcium ions to verify the function of calcium ions in inflammation. Overall calcium ion decrease exerts antiapoptosis and antioxidative stress functions: decreasing the transcriptional expression of CHOP, PERK, HSP70, Caspase-3, Caspase-7, Caspase-9, and Bax and increasing Bcl-2 and decreasing the total protein of HSP70 and increasing SOD1/2 expression (Figures 4(a)–4(c)).

Cell level of SOD was increased, and MDA was decreased in the LE group (Figure 4(d)). Flow cytometry verified that EGTA significantly decreased apoptosis and dead cells (Figures 4(e) and 4(f)). IF staining for ER-ID, ROS, IC-1, and DHR123 indicated ROS and ER stress, and mitochondrial stress significantly reduced in the LE group. (Figures 3(a) and 3(b)).

### 3.8. TRPA1 Inhibitor Significantly Reduced Periodontal Tissue Destruction in Periodontitis Mice

**Figure 5:**
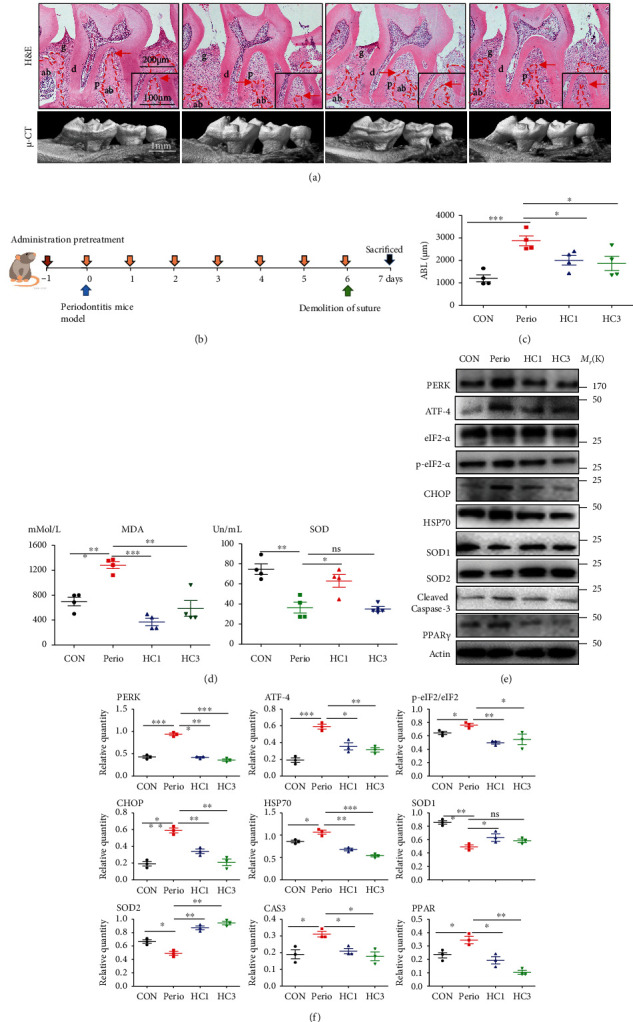
TRPA1 inhibitor significantly inhibited periodontal tissue destruction and oxidative stress in periodontitis mice. (a) Representative H&E staining and micro-CT images of mouse maxillary (*n* = 4): (1) negative control (healthy group intraperitoneally administered with the same solvent but without HC030031, CON); (2) periodontitis group (experimental periodontitis group intraperitoneally administered with the same solvent but without HC030031, Perio); (3) HC1 group (experimental periodontitis group intraperitoneally administered with 10 mg/kg HC030031, HC1); (4) HC3 group (experimental periodontitis group intraperitoneally administered with 30 mg/kg HC030031, HC3). (b) Detailed schematic diagram of the animal experiment process. (c) Quantitative statistical analysis of alveolar bone loss in micro-CT (*n* = 4). (d) Total SOD and MDAs of mouse serum (*n* = 4). (e, f) Western blot analysis and semiquantitative statistical analysis of mouse gingiva (*n* = 4). ABL: alveolar bone loss. Data analysis was performed by using one-way ANOVA (^∗^*P* < 0.05, ^∗∗^*P* < 0.01, and ^∗∗∗^*P* < 0.001). Error bars represent mean ± SEM.

To explore the potential application of TRPA1 inhibitor in periodontitis treatment, HC030031 was intraperitoneally administrated in experimental periodontitis mice (Figure 5(b) and Fig. S2). H&E staining revealed that periodontal tissue destruction was less in the HC1/3 group compared with the Perio group: less alveolar bone loss and inflammatory cell infiltration, more regularly arranged periodontal ligament tissue and space (Figure 5(a)). Micro-CT indicated the absorption of buccal and lingual alveolar bone was quantitatively less in the HC1/3 group (Figure 5(c) and Fig. S3a). IHC staining showed that TRPA1 was expressed in gingival, periodontal ligament, alveolar bone, and pulp tissue without specificity but significantly increased in the inflammatory intrasulcus epithelium (Fig. S2). Total CRP was higher in the periodontitis group and was lower in the HC1/3 group (Fig. S3c).

**Figure 6:**
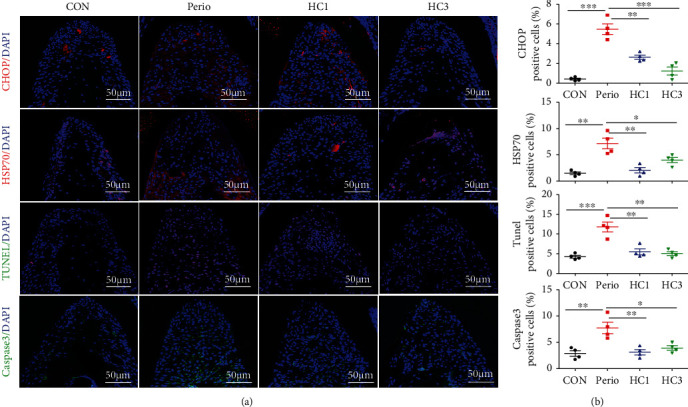
Oxidative stress and apoptosis levels in gingival tissue and in periodontal ligament tissue were significantly decreased. (a) Immunofluorescence staining of periodontal ligament tissue in mouse maxillary in CON, Perio, HC1, and HC3 groups (*n* = 4). (b) Semiquantification of immunofluorescence staining (*n* = 4). Data analysis was performed by using one-way ANOVA (^∗^*P* < 0.05, ^∗∗^*P* < 0.01, and ^∗∗∗^*P* < 0.001). Error bars represent mean ± SEM.

With the administration of HC030031, oxidative stress and apoptosis levels in gingival tissue and periodontal ligament tissue were significantly decreased in mice. SOD in serum was higher, and MDAs were decreased in the HC1/3 group (Figure 5(d)). In gingiva, cleaved Caspase-3, PPAR-*γ*, and HSP70 decreased significantly and SOD1/2 increased and PERK/eIF2*α*/ATF-4/CHOP signaling pathway was also significantly decreased (Figures 5(e) and 5(f)). In periodontal ligament tissue, the number of TUNEL-positive and cleaved Caspase-3-positive cells decreased significantly, and the fluorescence intensity of CHOP and HSP70 decreased (Figures 6(a) and 6(b)).

## 4. Discussion

Oxidative stress and subsequent apoptosis appeared earlier in periodontitis initiation [11] and sustained at a high level during periodontal development [16], which were consisted with unbalanced periodontal homeostasis resulting in periodontal tissue loss [15, 20]; thus, therapeutic targets decreasing oxidative stress and apoptosis were promising in periodontitis. Identically, in our study, higher levels of oxidative stress and apoptosis markers were observed in periodontal ligament tissues or cells from clinical samples or periodontitis mouse models and in *P.g.*LPS-induced inflammatory PDLCs in vitro. We found protecting periodontal tissues or cells from oxidative stress and apoptosis by blocking TRPA1 activation in inflammation significantly reduced periodontal tissue loss among mice.

**Figure 7:**
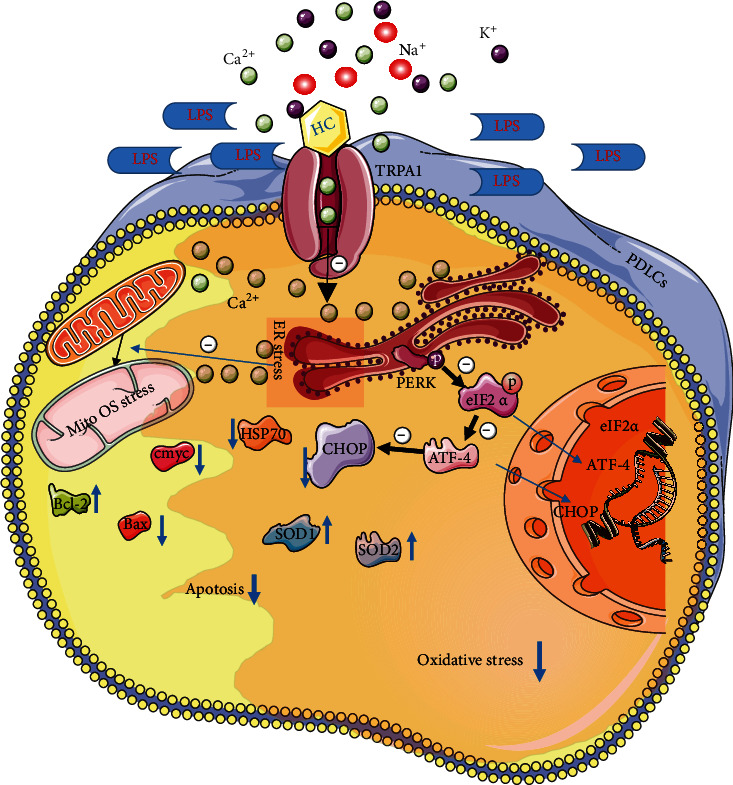
Schematic diagram of the mechanisms of TRPA1 in periodontitis. TRPA1 inhibition ameliorates periodontitis by reducing oxidative stress and apoptosis via PERK/eIF2*α*/ATF-4/CHOP signal pathway in inflammation.

As one of the superfamilies of TRP cation channels, TRPA1 has been reported to involve in inflammation and exert anti-OS and anti-OS-induced apoptosis effects in diverse diseases [23, 48, 49]. In the neural system, TRPA1 inhibition significantly decreased the intracellular Ca^2+^ concentration and attenuated demyelination by reducing the apoptosis of mature oligodendrocytes via antiapoptotic pathways (mitogen-activated protein kinase pathway, as well as transcription factors c-Jun and Bcl-2/Bak) [50]. In nonneural cells, TRPA1 inhibitors also decrease oxidative stress, inflammation, ER stress, mitochondrial dysfunction, and apoptosis and further lower the levels of the proinflammatory cytokines IL-1, IL-6, and TNF*α* [32]. Ca^2+^ influx and related proteins or subsequent organelle dysfunction were reported to be involved in TRPA1-related OS and apoptosis process [23–25, 27–29], but no more precious molecular mechanisms related were further defined. Namely, our study demonstrated its effect on reducing oxidative stress, apoptosis, ER stress, and mitochondrial dysfunction in LPS-induced inflammatory PDLCs via PERK/eIF2*α*/ATF-4/CHOP signaling pathway (Figure 7). Moreover, it significantly prevented periodontal tissue loss during periodontitis in vivo without the influence of mouse body weight, serum CRP, and organ damage (Fig. S3 & S4), indicating its potential therapeutic effects in periodontitis. Currently, TRPA1 inhibitors have been applied in the treatment of respiratory disorders in clinical trials [24, 25]. Many new inhibitors of TRPA1 still are in development with better properties. Due to the variety of inhibitors and different characteristics, it is still necessary to further study which inhibitors play roles in the treatment of related diseases. At the same time, the combination of appropriate biological materials with many insoluble TRPA1 inhibitors is also valuable for clinical transformation.

As the most Ca^2+^-permeable in TRP superfamily channels, TRPA1 can be activated by multiple factors, including Ca^2+^, pH, reactive oxygen, inflammatory factors, LPS, nitrogen and carbonyl compounds [51]. This study proved a significant activation and increase of TRPA1 in P-PDLC/Ts from periodontitis and in LPS-induced inflammatory PDLCs but not in the HC pretreatment group. The increased content of TRPA1 receptor also plays an important role in perturbing intracellular calcium concentration, while the reason how the content of TRPA1 increases was rarely known. Dubiquitinating induced by the tumor suppressor protein CYLD can increase TRPA1 protein [52]. The activation of the intracellular energy sensor AMPK (5′ AMP-activated protein kinase) is the other pathway to rapidly decrease membrane-associated TRPA1 [53]. Disruption of lipid rafts or the depletion of cholesterol in plasma membrane is also a mechanism to mediate TRPA1 content and TRPA1-mediated responses [54]. Vesicle fusion (named TRPA1 trafficking later) is a quite important mechanism to maintain the functional expression of TRPA1 in plasma membrane in inflammation and OS thus allowing a rapid activation to acute stimuli [55]. It was triggered by localized influx of Ca^2+^ and relayed on soluble N–ethylmaleimide-sensitive factor attachment protein receptor- (SNARE-) mediated vesicle transport [55], but the mediators and mechanisms involved remained largely unknown. Our study found that LPS, which can insert and induce mechanical perturbations in the plasma membrane to activate TRPA1 [56], significantly elevated TRPA1, although further studies are necessary to elucidate the mechanism of the increase of TRPA1 content or TRPA1 trafficking. This study also found that the pretreatment of the agonist (HC030031) did not influence TRPA1 expression in inflammation, it might be related with the agonist's electrophilic nature which determines TRPA1 trafficking [57]. TRPA1 trafficking attracted attention due to drug disrupting lipid rafts that could potentially mediate TRPA1 trafficking and substance function [54]. Thus, the cholesterol-TRPA1 interaction is worth studying, and it would be attractive to verify whether the combination of cholesterol-reducing therapies using statins and TRPA1 inhibition would be more perfect in inflammation and OS.

Ca^2+^ is a versatile and ubiquitous second messengers, and Ca^2+^ changes play an interactional role in multiple biological processes [58]. Cytosolic Ca^2+^ increase is always related with increased total ROS levels, cell apoptosis, energy metabolism dysfunction, ER stress, and mitochondrial dysfunction, and these processes will interactively further induce cytosolic Ca^2+^ increase via regulating Ca^2+^ gate by promoting the Ca^2+^ leaking from the plasma membrane or ER [59, 60]. Consistent with previous studies, we found increasing intracellular Ca^2+^ concentration induced by TRPA1 activation in inflammation in this study, which is in accordance with high levels of OS markers, apoptosis, ER stress, and mitochondrial dysfunction and with TRPA1 inhibitor, and the above cell functions significantly decreased combined with decreased intracellular Ca^2+^ concentration. In addition, we also used EGTA calcium chelating agent to reduce calcium ion concentration at the cell overall level and we wanted to observe how PSLCs would respond in inflammation under the condition of low calcium ion concentration. Interestingly, extracellular Ca^2+^ changes also significantly reduced cell apoptosis and oxidative stress. Although the precious molecular mechanisms should be further studied to proclaim the relationship between them, lower intracellular calcium concentrations were associated with lower levels of oxidative stress and apoptosis in inflammation.

ER stress occurs in severely impaired protein biosynthesis, secretion, folding, and cell death pathways [61]. In diver diseases, ER stress is often involved and plays double-edged sword role and even falls into a vicious cycle with excessive oxidative stress [62–64]. Oxidative stress interferes with the redox state of ER and results in ER stress and mitochondrial dysfunction and promotes the interaction between inflammation and apoptosis [65]. Our results demonstrated highly expressed ER stress markers such as CHOP and HSP70 in P-PDLCs/Ts and in LPS-induced inflammatory PDLCs and in periodontitis mice; thus, ER stress was involved in periodontitis. And the results of TEM and immunofluorescence indicated that ER morphology and size were significantly decreased after pretreated with TRPA1 inhibitor, which indicated that ER stress was implicated. Three ER transmembrane sensors are responsible for the delivery of UPR signal pathway activation in ER stress: inositol-requiring enzyme 1 (IRE1), protein kinase R-like endoplasmic reticulum kinase (PERK), and activating transcription factor 6 (ATF6) [66, 67]. PERK is responsible for resistance to oxidative stress to preconditioned ER stress [68]. PERK hyperactivation can inhibit antiapoptotic Bcl-2 expression by upregulating CHOP [69], a well-known mediator of ER stress-mediated cell death in multiple cell types [70]. PERK and its downstream factor and activating transcription factor 4 (ATF-4) are essential in ER process [66], and severe hypoxia can activate ER stress including PERK/eukaryotic initiation factor 2*α* (eIF2*α*) pathway [68]. In this study, PERK/eIF2*α*/ATF-4/CHOP signaling was detected and significantly activated in inflammation and was significantly downregulated by TRPA1 inhibitors (Figure 7). Moreover, with the treatment of PERK and CHOP inhibitors, cell apoptosis and OS were significantly decreased companioned with the significant ER stress decrease. This provides more accurate regulating information about the signaling pathways. As the number of compounds used for ER stress response increases in the laboratory, there is a strong interest in modifying these compounds or related compounds in clinical trials [71, 72], while efforts to inhibit ER stress response must be met with caution as this may be required to maintain homeostasis in some diseases.

## 5. Conclusions

In conclusion, TRPA1 was highly related to periodontitis, and the oxidative, apoptotic levels and TRPA1 expression were obviously higher in P-PDLC/Ts from clinical periodontitis patients, inflammatory PDLCs in vitro, and periodontitis mice in vivo. TRPA1 inhibitor significantly exerted antioxidative and antiapoptotic functions in inflammatory PDLCs via inhibiting ER stress by downregulating PERK/eIF2*α*/ATF-4/CHOP pathways. In vivo, less periodontium destruction, oxidative stress, and apoptosis in periodontium were observed after TRPA1 inhibitor administration; thus, TRPA1 was considered an attractive therapeutic target in periodontitis.

## Data Availability

The data of the findings in this study are available from the corresponding author upon reasonable request.

## Supplementary Materials

Supplementary Materials The supplementary material containing Table S1 and Figures S1-S4 and figure legends are available in supplementary files.
