# Supplementary material for: Inhibition of TRPA1 Ameliorates Periodontitis by Reducing Periodontal Ligament Cell Oxidative Stress and Apoptosis via PERK/eIF2α/ATF-4/CHOP Signal Pathway
Source: Oxid Med Cell Longev. 2022 Jun 10;2022:4107915. doi: 10.1155/2022/4107915 (PMC9205716; doi:10.1155/2022/4107915)
Supplement: Supplementary Materials — The supplementary material containing Table S1 and Figures S1-S4 and figure legends are available in supplementary files. [file 4107915.f1.pdf]

Supplementary Materials for

Inhibition of TRPA1 could ameliorates periodontitis by reducing Periodontal ligament cells oxidative stress and apoptosis via PERK/eIF2α/ATF-4/CHOP signal pathway

Qian Liu [1, 2, 3#], Shujuan Guo [1, 2, 3#], Yanli Huang[1, 2, 4], Xiuqun Wei[1, 2, 3], Li Liu[1, 2, 3], Fangjun Huo[1, 2], Ping Huang [1, 3], Yafei Wu [1, 3*], Weidong Tian [1, 4*]

Correspondence to: Yafei Wu, Weidong Tian; Email: yfw1110@163.com, drtwd@sina.com;

This supplementary file includes:

Table S1
Figure legends
Fig. S1
Fig. S2
Fig. S3
Fig. S4

Supplementary information

Table. S1 The primers sequences used in Real-time quantitative polymerase chain reaction

Gene		Sequence
Cas3	Forward	5′-CCAAAGATCATACATGGAAGCG-3′
	Backward	5′-CTGAATGTTTCCCTGAGGTTTG -3′
Cas9	Forward	5′-GACCAGAGATTCGCAAACCAGAGG-3′
	Backward	5′-AAGAGCACCGACATCACCAAATCC-3′
Bax	Forward	5′-CGAACTGGACAGTAACATGGAG-3′
	Backward	5′-CAGTTTGCTGGCAAAGTAGAAA-3′
Bcl-2	Forward	5′-GACTTCGCCGAGATGTCCAG-3′
	Backward	5′- GAACTCAAAGAAGGCCACAATC-3′
GRP78	Forward	5′-CAGTTGTTACTGTACCAGCCTA-3′
	Backward	5′-CATTTAGGCCAGCAATAGTTCC-3′
cymC	Forward	5′-GCCAATAAGAACAAAGGCATCA-3′
	Backward	5′-TTAAGTCTGCCCTTTCTTCCTT-3′
TRPM8	Forward	5′-TTCATCGTCTTCGCTTACTTCT-3′
	Backward	5′- GGCTTTTGTGTTGATCTTGACA-3′
TRPV1	Forward	5′-CTCACAGACAACGAGTTCAAAG-3′
	Backward	5′-CTTGTAGTAGCTGTCCGTGTAG-3′
TRPV4	Forward	5′-CCTACATCATCCTCACCTTTGT-3′
	Backward	5′-CTTCCTCAGGAATACGGGGAAG-3′
TRPA1	Forward	5′-CAGTGACCACAATGGCTGGACAG-3′
	Backward	5′-GTGCAGTGTTCCCGTCTTCATCC-3′
CHOP	Forward	5′-GAGAATGAAAGGAAAGTGGCAC-3′
	Backward	5′-ATTCACCATTCGGTCAATCAGA-3′
PERK	Forward	5′-CCAGTTTTGTACTCCAATTGCA-3′
	Backward	5′-CAGATACAGCTGGCCTCTATAC-3′
Cas7	Forward	5′-GCTGGGCAAATGCATCATAATA-3′
	Backward	5′-TGGCACAAGAGCAGTCATTATA-3′

HSP70	Forward	5′-GATATTGTGCAGTTGCCTACAG-3′
	Backward	5′-GGCTTCTTAAGAACACTTTCGG-3′

Supplementary information

Figure legend

Fig. S1 Cell viability test with drug concentration

Periodontal ligament cells derived from healthy were used in these tests. CCK8 assay was used to detect cell viability under different concentration of HC030031, 4-PBA, GSK2656157, EGTA (*n* =3). 10uM HC030031, 500uM 4-PBA, 10uM GSK2656157, 500uM EGTA were used in this study. Data analysis were performed by using one-way ANOVA (*P < 0.05, **P < 0.01, ***P < 0.001). Error bars represent mean ± SEM.

Fig. S2 Periodontitis mice model and TRPA1 expression in periodontium

a, Representative Micro-CT images, H&E staining and immunohistochemical staining (red error indicated highly expressed TRPA1 proteins) of mice maxillary in health control and periodontitis group. b, Quantitative statistical analysis of alveolar bone loss in Micro-CT (*n* =5). ALB: alveolar bone loss. Data analysis were performed by using one-way ANOVA (*P < 0.05, **P < 0.01, ***P < 0.001). Error bars represent mean ± SEM.

Fig. S3 Quantitative analysis of alveolar bone loss and changes of CRP and body weight

a, Quantitative statistical analysis of alveolar bone loss in buccal or in palatal of Micro-CT in Con, Perio, HC1 and HC3 groups (n =4). b, Body weight change of mice in Con, Perio, HC1 and HC3 groups (*n* =4). c, serum CRP levles change of mice in Con, Perio, HC1 and HC3 groups (*n* =4). Data analysis were performed by using one-way ANOVA (*P < 0.05, **P < 0.01, ***P < 0.001). Error bars represent mean ± SEM.

Fig. S4 H&E staining of mice organs

Representative H&E staining images of mice organs (heart, liver, spleen, lung, kidney) in Con, Perio, HC1 and HC3 groups.

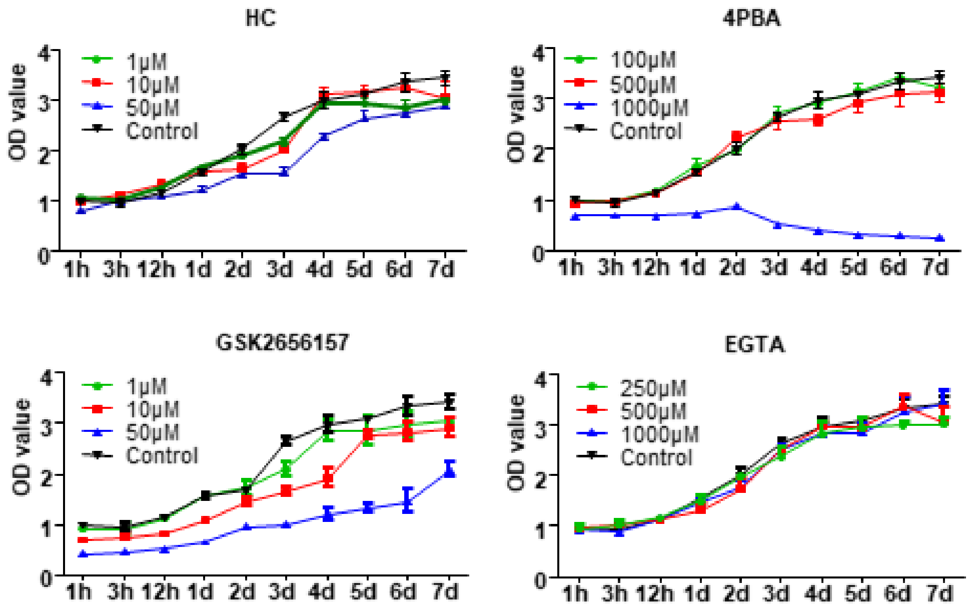

HC
1μM
10μM
50μM
Control
OD value
0
1
2
3
4
1h 3h 12h 1d 2d 3d 4d 5d 6d 7d
4PBA
100μM
500μM
1000μM
Control
OD value
0
1
2
3
4
1h 3h 12h 1d 2d 3d 4d 5d 6d 7d
GSK2656157
1μM
10μM
50μM
Control
OD value
0
1
2
3
4
1h 3h 12h 1d 2d 3d 4d 5d 6d 7d
EGTA
250μM
500μM
1000μM
Control
OD value
0
1
2
3
4
1h 3h 12h 1d 2d 3d 4d 5d 6d 7d

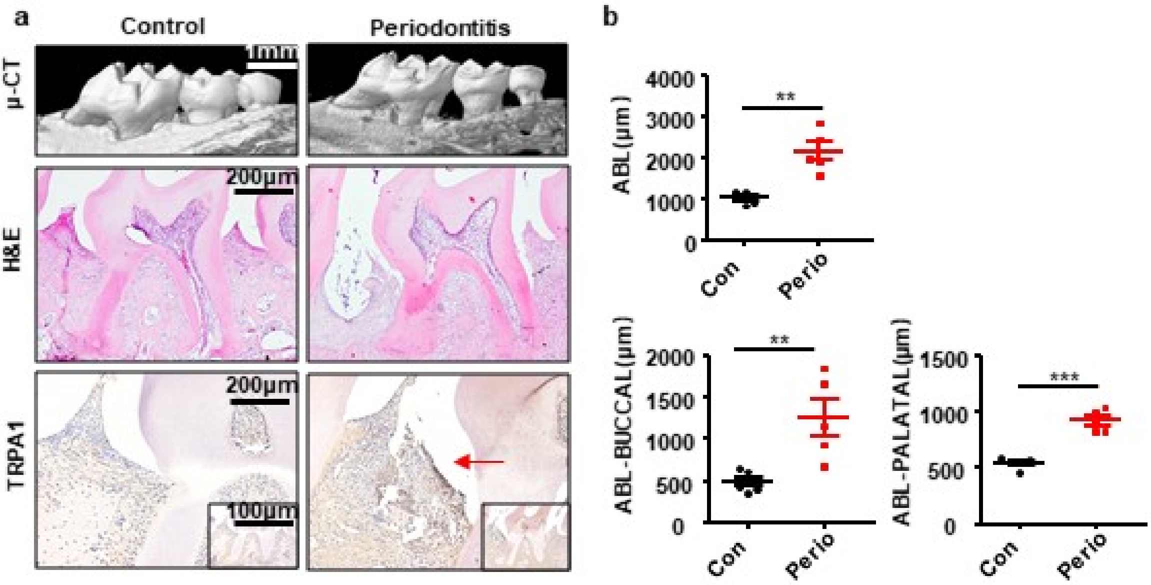
a
Control
Periodontitis
μ-CT
1mm
H&E
200μm
TRPA1
200μm
100μm
b
ABL(μm)
4000
3000
2000
1000
0
**
Con
Perio
ABL-BUCCAL(μm)
2000
1500
1000
500
0
**
Con
Perio
ABL-PALATAL(μm)
1500
1000
500
0
***
Con
Perio

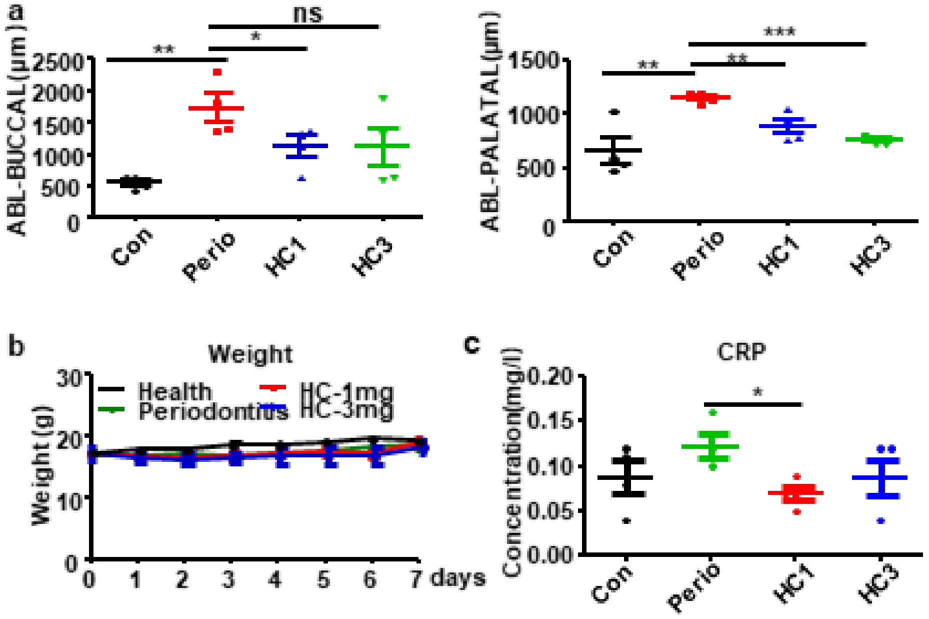

a
ABL-BUCCAL(μm)
ABL-PALATAL(μm)
Con
Perio
HC1
HC3
ns
b
Weight
Weight (g)
Health
Periodontitis
HC-1mg
HC-3mg
days
c
CRP
Concentration(mg/l)

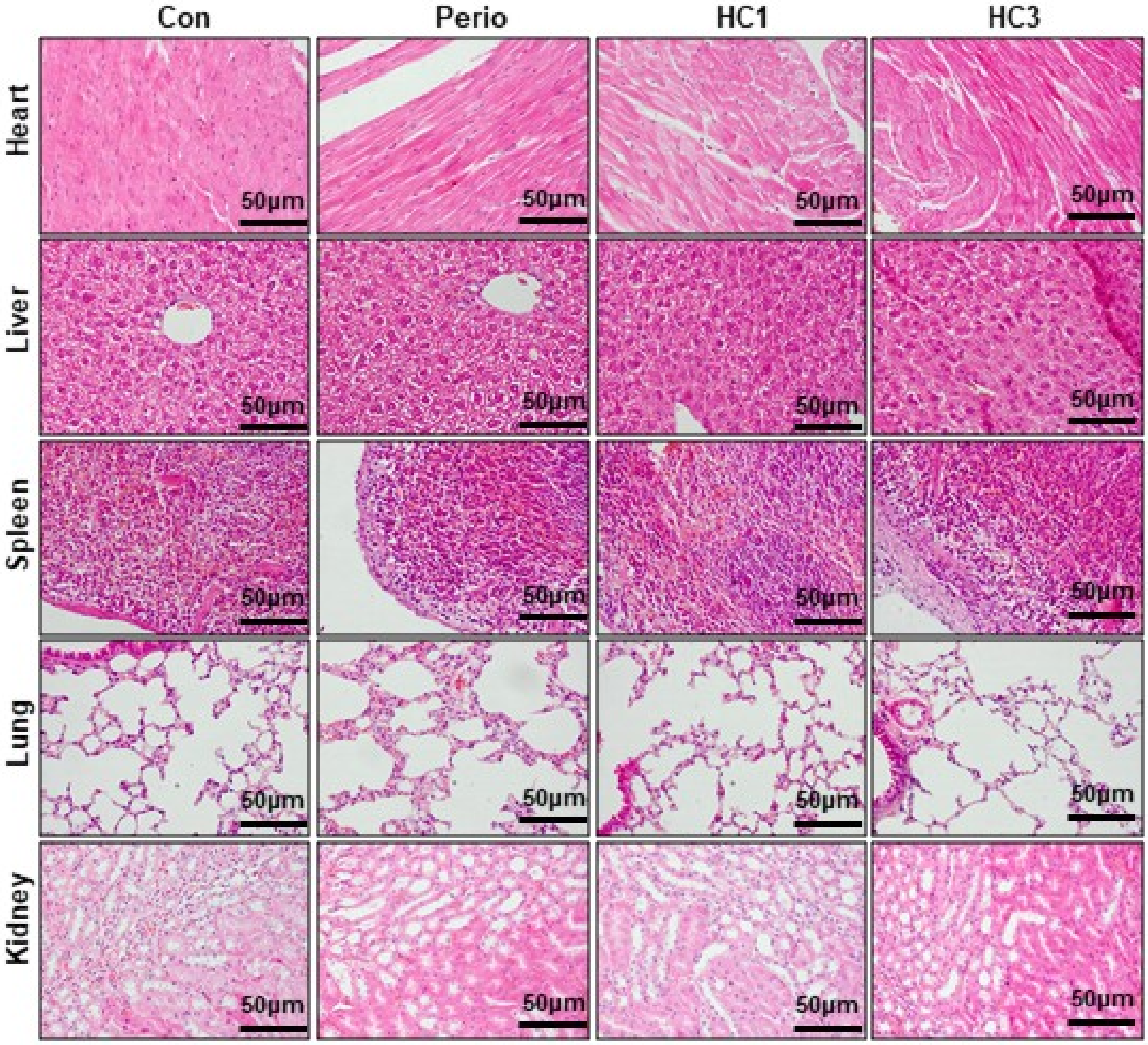

Con
Perio
HC1
HC3
Heart
Liver
Spleen
Lung
Kidney
50μm
